# Supplementary material for: The impact and cost of reaching the UNAIDS global HIV targets
Source: PLOS Glob Public Health. 2026 Oct 20;6(10):e0007360. doi: 10.1371/journal.pgph.0007360 (PMC13682660; doi:10.1371/journal.pgph.0007360)
Supplement: Supplementary file 2 [file pgph.0007360.s002.docx]

Supplemental Text: Resource needs estimates to increase access to justice for HIV vulnerable populations in low- and middle-income countries

**Scope and rationale**

The interventions including in this component are aligned with the UN resolutions and political declarations on access to justice and legal reforms. Meta-analyses and studies on the impact of interventions addressing legal barriers, promoting decriminalization and fostering legal empowerment of key populations. According to this information, decriminalization could prevent as many as 500,000 new infections among key populations from 2020-2030. Not surprisingly, global strategies from UNAIDS, Global Fund and PEPFAR also converge in both the narrative and recommended interventions to increase service uptake and reduce vulnerability, abuses and violence towards key populations.

A challenging legal environment, with restrictive and unequal laws, could impede the work of HIV providers and may discourage criminalized or stigmatized populations from seeking help. A robust legal framework would ensure non-discrimination for individuals living with HIV and those in high-risk groups, safeguarding them from biased treatment within health and social services. In response to both harmful and protective laws, it is important to ensure that key populations are well informed about their rights, that legal services are available to them to get legal counseling, filing complaints and seeking redress. Judges and magistrates, police officers and prison guards might not be aware of legal provisions, challenged laws, strategic litigation outcomes and so forth. Duty bearers need updates on the status of key populations as right holders.

We have selected interventions for access to justice and law reform that are based on UN resolutions and political declarations, meta analyses and studies identified the effectiveness of different interventions, and programmatic priorities in Global Strategies of UNAIDS, Global Fund and PEPFAR. These interventions in Table A. Recommendations from the UN resolutions and political declarations are presented in Table B. Table C summarizes the evidence on the impact of these interventions. Table D summarizes the global strategies referring to access to justice.

**Table A. Selected interventions for Access to Justice and Law Reform**

| **Intervention** | **Scope and Description** |
| --- | --- |
| Legal literacy (“Know your rights”) | • Legal/patients’ rights literacy trainings for women, girls and other vulnerable and key populations;  • Establishment of crisis response mechanisms to prevent abuse, including gender-based violence. |
| HIV and HIV/TB-related legal services | • Legal information, referrals, advice and representation related to HIV and HIV/TB, including through peer paralegal community systems;  • Legal services and counselling for key populations ; support through arbitration, dispute settlement mechanisms;  • Support to community forms of dispute resolution, including engagement of traditional leaders and customary law in support of people affected by HIV and HIV/TB; strategic litigation. |
| Sensitization of law-makers and law-enforcement agents | • Information and sensitization programs for parliamentarians, Ministries of Justice, judges, prosecutors, police and traditional and religious leaders on legal, health and human rights aspects of HIV and HIV/TB, including gender-and age-based discrimination and inequity and on violence prevention, intimate partner violence and their relation to HIV;  • Facilitation of discussions among service providers and law enforcement officers to gain police support for health programs;  • Training of prison personnel (both in prisons for women and men) on public health, human rights and HIV and HIV/TB responses; • HIV and HIV/TB in the workplace programs for law-makers and enforcers. |
| Improving laws, regulations and polices relating to HIV and HIV/TB | • Assessing impact of policies/practices on informed consent and confidentiality on access to services;  • Legal Environment Assessments, and community-based monitoring of laws and their implementation in terms of their impact on health and access to services;  • Advocacy and mobilization for law and policy reform to increase access to services. |

**Table B. Recommendations from UN Resolutions and Political Declarations on Access to Justice and Legal Reforms**

| Reference | Statement | Recommendations |
| --- | --- | --- |
| A/60/L.57  (June, 2006) | Political Declaration on HIV/AIDS (no. 15, 24, 29) | Committing to overcoming legal, regulatory or other barriers that block access to effective HIV prevention and treatment; enacting legislation to eliminate all forms of discrimination and exclusion. |
| A/HRC/32/31 (2016) | Human Rights Council Resolution on Civil Society Space (no. 7) | Urges States to ensure access to justice, and accountability to end impunity for human rights violations and abuses against civil society actors, including amendment of laws and policies |
| E/2019/74 (2019) | ECOSOC Resolution on UNAIDS (No. 33, 35, | Recognizes UNAIDS work promoting the removal of discriminatory laws and the creation of an empowering legal environment, to accelerate progress towards the SDGs |
| A/HRC/56/20 (2021) | HRC Resolution on Human Rights in the Context of HIV and AIDS (No. 9) | Calls upon States to end impunity for abuses against people at risk or affected by HIV, by ensuring access to justice, legal literacy, access to legal representation and training of judges, law enforcement officers and other duty-bearers. |
| A/RES/76/141 (2022) | Resolution on Violence against Women Migrant Workers | Facilitating access to justice, reforming harmful laws, enhance or develop protective frameworks. |

**Table C. Meta-Analyses and studies providing evidence-based interventions to increase access to justice, removing harmful laws and creating protective legal frameworks**

| Reference | Study |
| --- | --- |
| Stangl, Ann L et al (2022) | Removing societal and legal impediments to HIV response: an evidence based framework for 2025 and beyond. <https://journals.plos.org/plosone/article?id=10.1371/journal.pone.0264249> |
| USAID Open Doors (2017) | Understanding legal barriers to accessing HIV/AIDS services by key populations: findings from expert panel meetings in Zambia. <https://www.fhi360.org/wp-content/uploads/drupal/documents/resource-zambia-open-doors-expert-panel-report.pdf> |
| Goodwing L & V Maru (2017) | What do we know about legal empowerment? Mapping the evidence. Hage Journal on the Rule of Law. <https://link.springer.com/article/10.1007/s40803-016-0047-5> |
| Jones, L et al (2011) | Costing human rights and community support interventions as part of Universal Access to HIV treatment and care in a Southern African setting. <https://www.ncbi.nlm.nih.gov/pmc/articles/PMC3531822/> |
| Shannon et al. Lancet 2015; 385:55–71 | Modeling of the effects of decriminalization on incidence among FSW in Vancouver, Mombasa and Bellary showed 40% reduction in new infections  . |
| Borquez et al., Lancet Public Heal Published Online First: 2018. | Modeling of the effects of decriminalization on incidence among PWID in Mexico showed 21% reduction in new infection  doi:10.1016/S2468-2667(18)30097-5 |

**Table D. Priority Areas on Access to Justice and Legal Reform in Global Strategies**

| Source | Global Strategy | Targets and Interventions |
| --- | --- | --- |
| UNAIDS | 2021-2026 Strategy – Results Area 6: KP Enjoy Human Rights | Priority Actions: Removing punitive laws and policies, enforce protective and enabling legislation. Reform public health and law enforcement practices hampering the HIV response; increase meaningful access to justice and accountability for HIV-related HR violations; legal literacy, legal support and representation. |
| Global Fund | Removing human rights related barriers to HIV services – 2023-2025 <https://www.theglobalfund.org/media/12445/core_removing-barriers-to-hiv-services_technicalbrief_en.pdf> | Priorities for investment include: 3.2.3 Legal literacy; 3.2.4 increasing access to justice; 3.2.5 ensuring rights-based law enforcement practices; 3.2.6 improving laws, regulations and policies related to HIV and HIV/TB; 3.2.7 reducing HIV-related gender discrimination, harmful gender laws and violence. |
| PEPFAR | 5-year strategy Focus area 6: dismantling structural barriers to HIV care  <https://www.state.gov/wp-content/uploads/2022/11/20221107-DRFAT-PEPFAR-Strategy_For-Comment_CLEAN.pdf> | Coordinated with UNAIDS and Global Fund. Working with partners to address punitive laws and promoting adoption of enabling policies. |

**Population groups**

There are two types of population groups to estimate the scale of interventions and their increase over time: key population groups of the HIV response, and justice operators and law enforcers. The Goals based projections of key population groups by country, produced by Avenir Health, were utilized to project quantity of services of legal literacy, legal representation and legal counselling.

The number of judges and magistrates, and the number of law enforcers by country was obtained from data.UNODC.org, “Criminal Justice System: Professional judges or magistrate personnel, counts and rates per 100,000 population”. For those countries were rates were available, the total population projections were used to obtain the projected number of judges and magistrates up to 2040. In countries where rates were not available, the missing values were attributed according to quartiles of GDP in international dollars. The source for police forces and prison staff was also based on data from data.UNODC,org. The target populations for each intervention are given in Table E.

**Table E. Target Populations**

| **Programme** | **Activity** | **Quantification** |
| --- | --- | --- |
| Legal literacy | Community based paralegal network and human rights defenders | No. of networks |
|  | HIV Hotline / Online counseling | No. of months |
|  | Awareness rising campaign by paralegals | No. of sessions |
| Legal services | Strategic litigation fund |  |
|  | Training pro bono lawyers (KP Plaintiffs) | No. of lawyers |
|  | Training and sensitizing public defenders | No. of defenders |
| Training officers | HIV and Human Rights for police officers | No. of police officers |
|  | HIV & HR for Penitentiary officers | No. of prison guards |
| Law Reform | Advocacy subgrants for CLOs | No. of laws to reform |
|  | Coalitions for social mobilisation | No. of laws / campaigns |
|  | Developing proposals for law reform | No. of laws |

**Country stratification**

The country stratification was made on the basis of four categories:

a) *Income group*: Using the World Bank Classification, the categories of low, lower-middle and upper middle income were utilized to stratify countries. The provision costs were adjusted according to purchasing power of the local currency units.
b) *Epidemic level*: The epidemic level was divided in three categories: low, concentrated and generalized. The targets of the interventions were then adjusted to prioritize generalised epidemics.
c) *Population density*: For stratification purposes, population density was categorised in low, moderate and high density. The number of shops, online/hotline counselling increased when density was lower (higher dispersion).
d) *Legal environment*: based on the index on enabling environment, the component on legal environment was used as stratification variable in three categories: adverse, moderate and favourable. The number of activities to foster awareness and reforming laws and policies was higher in countries with adverse legal environments.

The stratifications are shown in Figure A.

**Fig A. Distribution of countries by category**

Countries included in the resource needs estimates by income group and HIV prevalence levels

|  | Total |
| --- | --- |
| Concentrated Low income | 8 |
| Concentrated Lower middle income | 4 |
| Concentrated Upper middle income | 6 |
| Generalised Low income | 8 |
| Generalised Lower middle income | 6 |
| Generalised Upper middle income | 5 |
| Low Low income | 15 |
| Low Lower middle income | 36 |
| Low Upper middle income | 23 |

The data sources used to develop the cost estimates are shown in Table F.

**Table F. Data sources and adjustments for cost estimates**

| Legal literacy Trainings; Human rights and HIV trainings for Justice Operators and Law Enforcers | Population group sizes by Avenir  Face-to-face training / Costing studies in 18 countries, updated by inflation rate  Costs (and effectiveness) of online training : “eLearning and eMarketing costs” by Meinert E, J Eerens et al (2021) JMIR Med Educ 2021 Mar 11;7(1):e13681; Munroe, Schackman, Madkins et al (2025) Comparative cost of implementing an Evidence-Based online prevention program: Direct-to-Consumer Marketing vs Community-Based Organization Recruitment; Huang, Marlin et al (2015) P17.09 Cost-effectiveness of HIV self-testing promotion through Grindr™, a smartphone social networking application  Based on UNOC police personnel, judges and magistrates for some countries, and GDP PPP , rates of law enforcers and justice operators were estimated and population sizes projected. |
| --- | --- |
| Legal services | Cost of hotline centres and legal aid clinics / Costing studies in 18 countries, updated by inflation rate; Global Fund Baseline assessments to scale up programmes to reduce rights related barriers – updated by inflation rate; Legal AID Clinic costs: UNDP Strategy for India.  Social media campaigns: Grov, Westmoreland, Carneiro et al (2020) Maximizing response rates to ads free at home HIV testing on men-for-men geosocial networking app. |
| Legal reform | Costing studies in 18 countries, updated by inflation rate |

Cost Assumptions to Reach Targets

Interventions costs were adjusted for each income/epidemic category are shown in Tables G (legal literacy), H (human rights training), I (legal services) and J (law reform).

**Table G. Adjustments to Factor in HIV Prevalence: Legal Literacy**

| **Delivery Modalities** | | **Stratification Criteria for Quantitative Targets** | | | | | **Total Cost Adjustment Based on HIV Prevalence** | | |
| --- | --- | --- | --- | --- | --- | --- | --- | --- | --- |
| No. | Activity | LIC | LMIC | UMIC | High Density | Adverse Legal Environment | Low prevalence | Concentrated | Generalised |
| 1 | Face-to-face training | 40% | 20% | 20% | 40% | 40% | 25% | 50% | 100% |
| 2 | Asynchronous online | 40% | 20% | 20% | 40% | 40% | 25% | 50% | 100% |
| 3 | Mobile learning | 20% | 60% | 60% | 20% | 20% | 25% | 50% | 100% |
| *LEI supersedes Density supersedes Income Group. Group 1= LIC or HIGH Density; Group 2 = LMIC or Adverse LEI; Group 3: UMIC, not high density no adverse LEI* | | | | | | |  |  |  |
| *Online courses and social media are preferred when meeting is unsafe, pop density is low and the country has adequate integration and connectivity (UMIC).* | | | | | | |  |  |  |

**Table H. Adjustments to Factor in HIV Prevalence: Human Rights Training for Law Enforcers and Justice Operators**

| **Delivery Modalities** | | **Target** | **Environment** | | **Total Cost Adjustment Based on HIV Prevalence** | | |
| --- | --- | --- | --- | --- | --- | --- | --- |
| No. | Activity |  | Adverse EEI | Adverse LEI | Low prevalence | Concentrated | Generalised |
| 1 | Law Enforcers | 30% | 35% | 40% | 25% | 50% | 100% |
| 2 | Asynchronous online | 30% | 35% | 40% | 25% | 50% | 100% |

**Table I. Adjustments to Factor in HIV Prevalence: Legal Services**

| **Id_Act** | **Activity** | **Target** | **Quanta** | **Base** | **Adverse EEI** | **Adverse LEI** | **Modality** | **Low** | **Moderate** | **High** |
| --- | --- | --- | --- | --- | --- | --- | --- | --- | --- | --- |
| P3_A1a | Hotline Legal Advice Centre | 1 per 100k KP | 0,00001 | KP | 0,0033% |  |  | 25% | 50% | 100% |
| P3_A1b | Online Legal Advice Centre | 1 per 100k KP | 0,000004 | KP | 0,0010% |  | Online: LMIC-UMIC | 25% | 50% | 100% |
| P3_A2 | Legal assistance clinics | 1 per million KP | 0,0000010 | KP |  | 0,00014% |  | 25% | 50% | 100% |
| P3_A3 | Media campaigns | 1 per 10 million inhabitants | 0,0000001 | POP | 0,00002% |  |  | 25% | 50% | 100% |
| P3_A4 | Legal representation | 1 case per 20k KP | 0,00002 | KP |  | 0,0033% |  | 25% | 50% | 100% |
| T*argets are set at the level of countries with moderate or low environment index and favourable legal environment index. Where conditions are worse, targets increase to provide more services to the key populations.* | | | | | | | | | | |

**Table J. Adjustments to Factor in HIV Prevalence: Law and Regulatory Reforms**

| **Id_Act** | **Activity** | **Target** | **Quanta** | **Base** | **Adverse LEI** | **Low** | **Moderate** | **High** |
| --- | --- | --- | --- | --- | --- | --- | --- | --- |
| P4_1 | Law Monitoring | Annual Report | 1 | Country |  | 25% | 50% | 100% |
| P4_2 | Social Media Awareness Campaign | 1 |  | Country | 2 | 25% | 50% | 100% |
| P4_3 | Drafting Proposals | Annual | 1 | Country | 2 | 25% | 50% | 100% |
| P4_4 | Lobbying and Advocacy | 1 | 1 | Country | 2 | 25% | 50% | 100% |

**Main results**

The resulting estimates of resource needs by intervention are shown in Figure B. Figure C shows resource needs by epidemic type and Figure D shows resources needs by country income category.

**Fig B. Resource needs to access to justice and legal reform by intervention category**

Access to Justice and Legal Reform Resource Needs 2026-2031 - By component-

|  | Legal Literacy | Human Rights Trainings | Legal Services | Legal Reform |
| --- | --- | --- | --- | --- |
| 2026 | 445858017 | 39179161 | 73231887 | 7320161 |
| 2027 | 455337024 | 39647331 | 74796002 | 7320161 |
| 2028 | 464870738 | 40093264 | 75943826 | 7320161 |
| 2029 | 474302266 | 40517794 | 77165228 | 7320161 |
| 2030 | 483588077 | 40912484 | 78721749 | 7320161 |
| 2031 | 484579092 | 41792405 | 79049594 | 7320161 |

**Fig C. Resource needs to access to justice and legal reform by epidemic type**

**Fig D. Resource needs to access to justice and legal reform by income level**

**Access to Justice and Legal Reform: Resource Needs 2026 – 2031 by Income Level**

|  | Sum of 2026 | Sum of 2027 | Sum of 2028 | Sum of 2029 | Sum of 2030 | Sum of 2031 |
| --- | --- | --- | --- | --- | --- | --- |
| Low income | 180830153 | 186426265 | 191705740 | 197426349 | 203377059 | 204228155 |
| Lower middle income | 184438130 | 188474918 | 192596874 | 196228081 | 200022423 | 200951481 |
| Upper middle income | 200320943 | 202199334 | 203925374 | 205651019 | 207142988 | 207561614 |

**Validation of the results**

The categories, targets, activities and populations in this component were revised, commented and changes were suggested by the Equity in Rights for All (ERA) team at UNAIDS.

The unit and total costs obtained with the estimates were compared with the Global Fund records on country expenditure on access to justice and legal reform, and some adjustments were made whenever the expected expenditure were much higher than absorptive capacity of the countries.

Scope and limitations

The programmatic components are results-oriented and defined by main purpose. The databases for all territories and states were adjusted to low- and middle-income countries, including those categorised in these groups in 2020 and onwards. The costing follows a gradualist approach, scaling coverage and investments over time.

There are synergies between components and with societal enablers estimates. Both areas contribute to higher acceptation and integration.

The targets and resource needs for access to justice and legal reform developed in 2020 to be reached in 2025, were not adequately followed to document progress and focus on programmatic and financial gaps.

**Bibliography**

a) UNAIDS Guidance Note (2014). *Human Rights and the Law*. UNAIDS / JC2568 (English original, May 2014). UNDP / UNAIDS. <https://www.unaids.org/sites/default/files/media_asset/2014unaidsguidancenote_humanrightsandthelaw_en.pdf>
b) UNAIDS Toolkit (2009) *Scaling up HIV related legal services*. UNAIDS / UNDP / IDLO. <https://www.hivlawandpolicy.org/resources/toolkit-scaling-hiv-related-legal-services-idlo-unaids-undp-2009>
c) UNAIDS (2020). **2025 AIDS Targets. Target-setting for 2025 and resource needs and impact estimation for 2021–2030**. Strategic Information Department.
d) UNDP (2018) HIV and the Law. Supplement. Global Commission on HIV and the Law. Supplement. New York.
e) IDLO (2020) legal empowerment, social accountability and HIV prevention for young women and girls: lessons from Tanzania and Uganda. <https://www.idlo.int/publications/legal-empowerment-social-accountability-and-hiv-prevention-young-women-and-girls>
f) UNAIDS (2012), ‘Key Programmes to Reduce Stigma and Discrimination and Increase Access to Justice in National HIV Responses, Guidance Note’, Geneva: UNAIDS.
g) Schwartlander B et al (2011) Towards an improved investment approach for an effective response to HIV/AIDS. Lancet 2011; 377: 2031–41.
h) Sofia Gruskin, Kelly Safreed-Harmon, Tamar Ezer, Anne Gathumbi, Jonathan Cohen and Patricia Kameri-Mbote (2013). **Access to justice: evaluating law, health and human rights programmes in Kenya**. Journal of the International AIDS Society 2013, 16(Suppl 2):18726
i) Artur Domosławski (2011) **Drug Policy in Portugal The Benefits of Decriminalizing Drug Use**. Global Drug Policy Program. Open Societies Foundation.
j) Leo Beletsky, Rachel Thomas, Marina Smelyanskaya, Irina Artamonova, Natalya Shumskaya, Aijan Dooronbekova, Aibek Mukambetov, Heather Doyle, Rebecca Tolson (2012). **Policy reform to shift the health and human rights environment for vulnerable groups: The case of Kyrgyzstan’s Instruction 417**. Health and human rights, Volume 14, no. 2 December 2012
k) Tara SH Beattie, Parinita Bhattacharjee, BM Ramesh, Vandana Gurnani, John Anthony, Shajy Isac, HL Mohan, Aparajita Ramakrishnan, Tisha Wheeler, Janet Bradley, James F Blanchard, Stephen Moses (2010). **Violence against female sex workers in Karnataka state, south India: impact on health, and reductions in violence following an intervention program**. BMC Public Health 2010, 10:476.
l) Elena Argento , Sushena Reza-Paul , Robert Lorway , Jinendra Jain , M. Bhagya , Mary Fathima , S.V. Sreeram , Rahman Syed Hafeezur & John O'Neil. (2011) **Confronting structural violence in sex work: lessons from a community-led HIV prevention project in Mysore, India**, AIDS Care, 23:1, 69-74.
m) Global fund. **Modular Framework Handbook**, October 2019. (page 94). <https://www.theglobalfund.org/media/4309/fundingmodel_modularframework_handbook_en.pdf>
