# Supplementary material for: The impact and cost of reaching the UNAIDS global HIV targets
Source: PLOS Glob Public Health. 2026 Oct 20;6(10):e0007360. doi: 10.1371/journal.pgph.0007360 (PMC13682660; doi:10.1371/journal.pgph.0007360)
Supplement: Supplementary file 3 [file pgph.0007360.s005.docx]

Author’s Response

1. Please note that PLOS Global Public Health has specific guidelines on code sharing for submissions in which author-generated code underpins the findings in the manuscript. In these cases, we expect all author-generated code to be made available without restrictions upon publication of the work. Please review our guidelines at <https://journals.plos.org/globalpublichealth/s/materials-and-software-sharing#loc-sharing-code> and ensure that your code is shared in a way that follows best practice and facilitates reproducibility and reuse.

**Response**: There was no code generated for this manuscript. Two existing models were used: Goals and the Resource Needs Model. Both are freely available as mentioned in the manuscript.

2. Please send a completed 'Competing Interests' statement, including any COIs declared by your co-authors. If you have no competing interests to declare, please state "The authors have declared that no competing interests exist". Otherwise please declare all competing interests beginning with the statement "I have read the journal's policy and the authors of this manuscript have the following competing interests:"

**Response**: TP is a freelance consultant and received consulting fees from BioNTech, GSK, Pathfinder, John Snow Inc and UNAIDS through Impact Epilysis. TP received consulting fees from BioNTech, GSK and UCB. TP declares contracts with Impact Epilysis, BioNTech, GSK and UCB. MS received consulting fees from GSK and declares contracts with GSK. JS, DM and RV have declared that no competing interests exist.

3. Please amend your detailed Financial Disclosure statement. This is published with the article. It must therefore be completed in full sentences and contain the exact wording you wish to be published.

   a. State the initials, alongside each funding source, of each author to receive each grant.
   b. State what role the funders took in the study. If the funders had no role in your study, please state: “The funders had no role in study design, data collection and analysis, decision to publish, or preparation of the manuscript.”

**Response**: JS, TS, RV and MS received consulting fees from UNAIDS. DM is employed by UNAIDS. UNAIDS contributed to the design of the project, the review of data and assumptions, the review of the results, the decision to publish and the draft of the manuscript.

4. In the online submission form, you indicated that “Workbooks with all data and calculations are available from the authors.”.

   All PLOS journals now require all data underlying the findings described in their manuscript to be freely available to other researchers, either
   1. In a public repository,
   2. Within the manuscript itself, or
   3. Uploaded as supplementary information.

   This policy applies to all data except where public deposition would breach compliance with the protocol approved by your research ethics board. If your data cannot be made publicly available for ethical or legal reasons (e.g., public availability would compromise patient privacy), please explain your reasons by return email and your exemption request will be escalated to the editor for approval. Your exemption request will be handled independently and will not hold up the peer review process, but will need to be resolved should your manuscript be accepted for publication. One of the Editorial team will then be in touch if there are any issues.

**Response**: The Resource Needs Model, the unit cost database and all of the results are now available on-line at <https://rne.unaids.org/>. This has been added to the manuscript.

5. If the reviewer comments include a recommendation to cite specific previously published works, please review and evaluate these publications to determine whether they are relevant and should be cited. There is no requirement to cite these works unless the editor has indicated otherwise.

**Response**: We did cite the article recommended by the reviewer.

Additional Editor Comments (if provided):


[Note: HTML markup is below. Please do not edit.]

Reviewers' comments:

Reviewer's Responses to Questions

**Comments to the Author**

1. Does this manuscript meet PLOS Global Public Health’s [publication criteria](https://track.editorialmanager.com/CL0/https:%2F%2Fjournals.plos.org%2Fglobalpublichealth%2Fs%2Fcriteria-for-publication/1/010f019e82396676-3a8c68c8-906e-4de8-b20d-1427c471fc3a-000000/QUSIuX0xvpiadq27X623ex6k2OsDHI7KJGtCLw1c3Ks=258)? Is the manuscript technically sound, and do the data support the conclusions? The manuscript must describe methodologically and ethically rigorous research with conclusions that are appropriately drawn based on the data presented.

Reviewer #1: Yes

2. Has the statistical analysis been performed appropriately and rigorously?

Reviewer #1: N/A

3. Have the authors made all data underlying the findings in their manuscript fully available (please refer to the Data Availability Statement at the start of the manuscript PDF file)?

The [PLOS Data policy](https://track.editorialmanager.com/CL0/https:%2F%2Fjournals.plos.org%2Fglobalpublichealth%2Fs%2Fdata-availability/1/010f019e82396676-3a8c68c8-906e-4de8-b20d-1427c471fc3a-000000/dmyXSX-tXDMGUqu6Pzb29IxQspfrWyLtiguGEhgjc6M=258) requires authors to make all data underlying the findings described in their manuscript fully available without restriction, with rare exception. The data should be provided as part of the manuscript or its supporting information, or deposited to a public repository. For example, in addition to summary statistics, the data points behind means, medians and variance measures should be available. If there are restrictions on publicly sharing data—e.g. participant privacy or use of data from a third party—those must be specified.

Reviewer #1: Yes

4. Is the manuscript presented in an intelligible fashion and written in standard English?

PLOS Global Public Health does not copyedit accepted manuscripts, so the language in submitted articles must be clear, correct, and unambiguous. Any typographical or grammatical errors should be corrected at revision, so please note any specific errors here.

Reviewer #1: Yes

5. Review Comments to the Author

Please use the space provided to explain your answers to the questions above. You may also include additional comments for the author, including concerns about dual publication, research ethics, or publication ethics. (Please upload your review as an attachment if it exceeds 20,000 characters)

Reviewer #1: This manuscript reports the health impact and costs of reaching the UNAIDS global HIV prevention and treatment targets by 2030, based on the model projections. Global HIV epidemic control is heavily dependent on international and domestic resources, but more international donors are reducing their financial assistance in recent years, making it harder for counties to continue combating HIV epidemic. The authors’ efforts to demonstrate health impact and cost of achieving those targets could help understand resource needs and potential means to better allocate limited resources.

Major comments:

1. Modeling methods and perspective: The analysis was based on the Goals model, potentially a micro-simulation model of cohorts with various population characteristics. It was not clearly described in the methods, and too brief in the abstract. Although a previous paper was cited, a succinct description about the model and analysis platform/software would be helpful for readers to follow your analysis. The presentation was aggregated in that 2030 UNAIDS targets were presented based on 134 country-level results. So, it certainly involved several layers of aggregation, i.e., county-region-global. Overall resource allocation relates to global funding whereas the local resource mobilization should be based on country level data. This brings some confusion about analysis perspective, which guides to what costs to include or exclude in the analysis. Unit costs used in the analysis may not be a true reflection of overall resources needs, if the costs excluded country-level administrative overhead and donor overheads; it was not clear how those costs were handled in the analysis.

**Response**:

a. We slightly modified the description of the Goals model to provide more information.
b. The analysis was done at country level and then aggregated to the global level. We added emphasis on this approach in the Methods section. We also added a discussion of how unit costs might change in the new funding environment.

   2. Interventions and results: Not clear if it was an optimization model, and if the interventions can be prioritized, given the limited resources. Overall prioritization of the intervention from donors’ perspective may of interest, perhaps focus on key interventions showing high impacts. But country-level needs and domestic resource utilization and share of funding might lead to different prioritization. Although primary results were presented at a global level, you may focus on regional level results for details and stratifications (e.g., Figure 3); country-level results can be included in the appendix and would be helpful.

**Response**: This is not an optimization model because it attempts to estimate the resources that are needed rather than the best allocation of resources currently available. We added some text in the Discussion section to address this. Country-level results are available through the Resource Needs Model Online now referenced in the paper.

3. Uncertainty and sensitivity analysis: The modeling analysis and projections involved many input parameters and data/modeling assumptions; thus, uncertainty analysis should be an integral part of the presentation. The epidemiological and cost data were derived from various sources, and often they were assumed to be the same across counties and regions. Some basic sensitivity and scenario analyses may be used to address some of these issues.

**Response**: We have added ranges to the epidemiological indicators and added a paragraph at the end of the Results section with some sensitivity analysis on the unit costs.

Minor comments:

1. Line 26: including year of the Task Team, 2024 (?), would be useful.

**Response**: This has been added.

2. Line 28: Methods presentation was very brief; some details would help readers, not reading the full paper.

**Response**: We have expanded the Methods description in the Abstract.

3. Line 41-42: complex sentence, harder to read and understand what is being described.

**Response**: We have rewritten that sentence.

4. Line 43-44: just one sentence on cost projection! Need more balanced presentation, perhaps clear focus on resource/cost impacts. May separate US and $, or say US dollars?

**Response**: We have expanded the description of costing results.

5. Line 50-51: why separate $ from the number? Need consistency in presentation.

**Response**: This has been corrected.

6. Line: 74: Unusual to cite table in the introduction! It may be included in the methods.

**Response**: We prefer to keep the table on UNAIDS targets in the Background section since the methods describe how we estimated the costs and impact of reaching those targets. This paper is not about how the targets were set, but what they imply. So it does not seem right to put the targets in the Methods section.

7. Line 78: May start the sentence with 2024 Taks Team meeting, rather than describing previously published paper in the starting sentence! Methods section may be condensed, and description may be focused on main components of the model.

**Response**: We have revised the opening sentences as suggested and shortened the description of the enabling environment calculations.

8. Line 104-133: long description about gender-based violence, discrimination, access to justice, sounds very general, and may be moved to appendix. Perhaps interventions should be prioritized on testing, treatment, and viral suppression, the core components, although other social/cultural factors may play roles in successful implementation?

**Response**: The costs for these societal enablers is a unique feature of the UNAIDS resource needs estimates. In this exercise special effort was made to improve these estimates. We feel a full explanation of the approach does need to be in the body of the article.

9. Line 136: ‘above-site level’ cost? a new term, what does this include and how it was defined?

**Response**: We removed the term ‘above-site level costs’ and replaced with ‘health system strengthening’.

10. Line 138: ‘Ratios of support services to direct intervention spending’ sounds like a major part of the methods, but not clearly defined.

**Response**: We modified the sentence to be clear that these are percentages of direct costs calculated from National AIDS Spending Assessments.

11. Line 148: unit cost description could be condensed, and perhaps include input table; online appendices are helpful too.

**Response**: The unit costs are key to the resource needs estimates. We feel that is it important to give a full explanation here, in the body of the paper, to describe how we developed the database of unit costs.

12. Line 82: may cite Table or Figure with results.

**Response**: Done.

13. Line 130: ‘From 2010 to 2030’? Not clear why the projection had to go that far back!

**Response**: We use 2010 to 2030 because the UNAIDS targets are defined as 90% reductions in incidence and mortality from 2010 to 2030.

14. Line 218: ‘Commodities’ sounds very generic, can use specific term, e.g., tests, medicines/drugs, and materials?

**Response**: ‘Commodities’ refers to the list of commodities shown in Table 3. We have added some words to this sentence to refer the reader to the table.

15. Line 222: ‘Target needs’ sounds different from resource needs described above, and here you have regional stratification; as noted in the major comments, this could be a major thread to present results. Also, providing sub-headings for major sections might help guide readers.

**Response:** Sorry, we could not find the phrase ‘Target needs’. We have added sub-headings.

16. Line 268: a period missing after index.

**Response**: Corrected

17. Line 273-274: “foundation for countries to benchmark their current and projected
18. 274 domestic allocations”, this suggests country-level projections, and that may be included in the appendix. Also, this brings the question about mixing perspectives of donor vs countries domestic resource allocation, costs, and priorities.

**Response**: We have recently launched an online tool ‘Resource Needs Model’ that can be accessed through the UNAIDS aidsinfo.com website (under Financial Dashboard) that provides the country level data and estimates for all countries. We have added text to the Methods section to alert readers to this resource. The tool also allows programs to be costed using donor-based unit costs or domestic unit costs.

19. Line 295: very few references listed (n=11), undermining the available literature and contributions of others in the field! It shows the literature review on interventions, methods, economic analysis, etc., not be comprehensive!

**Response**: Most of the references are in the supplemental materials. We have added the most important sources of unit cost information to the main reference list.

20. Figures and captions not clear, e.g., Figure 2 has many acronyms not defined, to make them independent of the text.

**Response**: The caption for Figure 2 includes definitions of the acronyms. This is hard to see since the caption and figure are in separate files but in the published version they will be together.

21. The following pre-print article may be of interest, showing program-level impact of funding reduction.

**Response**: Thanks we have added this and two published studies as references for the potential impact of funding cuts.

Reference:
The impact of the United States foreign aid freeze on HIV service delivery in PEPFAR-supported countries: a facility-level analysis of 2024–2025 programme data
<https://www.medrxiv.org/content/10.64898/2026.04.17.26351143v1?ct=>
