# Supplementary material for: The impact and cost of reaching the UNAIDS global HIV targets
Source: PLOS Glob Public Health. 2026 Oct 20;6(10):e0007360. doi: 10.1371/journal.pgph.0007360 (PMC13682660; doi:10.1371/journal.pgph.0007360)
Supplement: Supplementary file 4 [file pgph.0007360.s006.docx]

# The impact and cost of reaching the UNAIDS global HIV targets

## Response to Reviewers, August 2026

**Reviewer #1:**

Revised manuscript improved greatly in terms of clarity and presentation, and reviewers’ comments are addressed.

Minor comments:
1. Figure labels were not clearly presented; e.g., Line 192-193, cited Figure 1a, 1b, but in Line 201, it was cited as Figure 1. Figure 2, presentation looks like a single bar, perhaps alternative presentation would be clearer for readers.

**Response**: Line 201 has been updated to refer to both Figures 1a and 1b. Figure 2 is a single stacked bar. We feel that this is a better format than a pie charts since many of the components are quite small in proportion to the total.

**Reviewer #2:**

Given the current HIV funding landscape, this is a timely study comparing the costs/resources needed and epidemiological impacts of two scenarios: 1) keeping all intervention coverage at 2023 constant levels versus 2) linear scale up of each intervention from 2025 to target value by 2030. Results from this study can enforce a call to action (i.e., that we still need mobilize additional resources including substantial increases in HIV funding in order to decrease HIV/AIDS incidence and related deaths).

The manuscript could benefit from several clarifications prior to publication as detailed below:

Major comments
2. The Methods section in the Abstract and main manuscript text could benefit from explicitly stating whether the study included LMIC only or LMIC and high income countries. In particular, in line 31, it seems like only 118 LMIC countries were included in the study; however, lines 47-48 quantify budget needed for high income countries as well. Further, lines 45-46 discuss the “resources needed to achieve the targets in all low- and middle-income countries (LMIC) would rise to US$21.9 billion by 2030); however, lines 47-48 state “of this [US$21.9 billion] amount…45% in upper-middle income countries.” It is unclear why the upper-middle income country budget would be reported as a proportion of the LMMIC budget. In line 101, it would be helpful to specify whether these 134 countries are LMIC or does it also include high income countries?

**Response**: All the financial results refer to 118 low- and middle-income countries (LMIC) which are disaggregated according to World Bank categories into low income, lower middle income and upper middle income countries. High income countries are not included. We added this clarification to the abstract.

3. The Methods would benefit from additional details about the Goals and Resource Needs Models. The manuscript currently states that these are “simulation models”. However, it is unclear whether these are microsimulation models, agent-based models, etc. Further, is it unclear whether these models are dynamic or static and whether indirect effects from the interventions were included in the models.

**Response**: We expanded the description of the Goals model.

4. Several sentences lack references (e.g., lines 66-67, lines 78-80, line 86, lines 120-121, lines 142-143, line 281). These line numbers are not an exhaustive list. The manuscript would benefit from the authors thoroughly checking and ensuring references are included where appropriate throughout the manuscript.

**Response**:

- The reference for lines 66-67 is given at the end of the paragraph.
- We added a reference to the target setting document (lines 78-80)
- The reference for line 86 is given at the end of the paragraph.
- Reference added for stigma and discrimination (lines 120-121)
- Reference added for national AIDS spending assessments (lines 142-143)
- Reference added for line 281.
- We have checked for other missing reference but believe that these are all.

5. Given donor funding cuts from PEPFAR and internationally, other modelling studies have modelled the impacts of reduced intervention coverage. (E.g., see: Ratevosian, J., Ngangula, P., & Tram, K. H. (2025). Modeling the fallout: projecting the global impact of donor funding cuts on HIV prevention, treatment, and care. Current Opinion in HIV & AIDS, 20(6), 621–631. <https://doi.org/10.1097/COH.0000000000000977>; Godbole, R., Wachinger, J., Nichols, C. et al. Shifts in PEPFAR-supported HIV treatment in 26 countries from 2024 to 2025. Nat. Health (2026). <https://doi.org/10.1038/s44360-026-00159-1>). The current manuscript could also benefit from modelling a decreased intervention coverage scenario in addition to the two scenarios modelled.

**Response**: We have cited several articles about the effects of the funding cuts in the discussion. But we do not believe that this article should also address that issue. This article is focused on the resource needs, to support resource mobilization efforts, and not on other scenarios of funding.

6. The Methods would benefit from more details in costs including: 1) specifying what year costs were reported in (e.g., 2026 USD); and 2) specifying whether discounting and/or inflation were considered.

**Response**: We added text to make it clear that all unit costs are in 2024 US dollars. No inflation was applied to future prices. We did update value from years before 2024 with the US GNP price deflator.

7. Lines 263-266 state that, “An increase of 20% in the cost of ARVs in 2030 across all countries would raise the total resource needs by 4%. A similar increase in the unit costs of service delivery for treatment or laboratory tests would increase total needs by 4% and 2% respectively.” Originally, I understood the 20% increase in the cost of ARVs in 2030 to be due to the increased number of people on ARVs. However, the wording in the second sentence seems to imply that it is the unit cost of ARVs that are increasing resulting in a 20% increase in the cost of ARVs. If this is true, it would be helpful to 1) specify the assumed proportion increase in ARV unit costs in the Methods and Data section; and 2) explain why unit costs of ARVs are expected to increase (e.g., is the use of newer patented ARVs expected to outweigh the increased use and availability of generic ARVs?).

**Response**: This is part of the sensitivity analysis, not a forecast of actual prices. The 20% refers to the unit cost. We have added language in that paragraph to clarify this point.

8. Some parts of the Discussion do not flow from the Results. For example, lines 276-277 state that “estimates [from the current study] update previous work that estimated that new infections would fall to 370,000 new infections and 250,000 AIDS deaths by 2025.” It would be helpful to mention what results from the current study these findings are being compared to and discuss what the key factors responsible for the differences in results between the current study and the previous study are? Another example is lines 281-282 which state that the “price reductions observed for ARVs during the 2021-2024 period.” This seems to contradict previous results stating an increase in ARV costs. Could authors clarify?

**Response**: The Results section explains that the current study projects new infections falling to 300,000 by 2030. Lines 276-277 compares that to the previous study when they were projected to fall to 370,000 by 2030. We updated the sentence to make that clear and describes the key reasons for the differences.

ARV costs increased as the number of people receiving treatment increased. However, the price of drugs per person-year of treatment dropped from 2021-2024.

9. While a couple of limitations are mentioned in the Discussion, the Discussion could benefit from a Limitations section that discusses model limitations, data limitations, limitations due to assumptions, etc., and the impact of these limitations on study findings in more detail.

**Response**: We added a paragraph in the Discussion providing more detail on the limitations.

Minor comments
10. Lines 25-26: Check grammar, capitalization, spacing.

**Response**: Thanks for catching that. That sentence has been corrected.

11. Line 46: LMIC could have been defined earlier in the abstract (i.e., line 31).

**Response**: We have now defined LMIC in the methods section of the abstract.

12. Lines 66-68: It is unclear whether these statistics are global, for low and middle income countries, or for another region.

**Regions**: We clarified that those statistics are for all countries.

13. Adding subheadings throughout the Methods would help improve readability.

**Response**: We have added four sub-headings: Epidemiological Impact, Resource Needs, Unit Costs and Coverage Targets.

14. Lines 143-145: Check grammar

**Response**: We have corrected the grammar.

15. Please ensure all acronyms (e.g., ARV) are defined upon first use.

**Response**: We have ensured that all acronyms are defined on first use.

16. Line 158: Define what year “current” is referring to in “current procurement prices.”

**Response**: We have change to ‘2025 procurement prices’

17. Lines 168-171: In addition to the absolute $ declined from current to 2030 prices, would be helpful to add a proportion (e.g., X% decline compared to current prices).

**Response**: Done

18. Throughout the Results, it would be helpful to indicate that the numbers in brackets are 95% CI (e.g., lines 191-192).

**Response**: Done

19. Line 194: Can authors confirm whether the decline from “40 (37-46) million to 39 (36-45) million” was a decline from 2023 to 2030?

**Response**: Yes, that is correct.

20. Lines 222-223 state that the “financial resources… were estimated to increase to just over $21.9 billion by 2030.” It is unclear what the comparator is (i.e., increase from when) and whether this number is referring to $21.9 billion/year for all included countries or $21.9 billion cumulative for all included countries.

**Response**: We have modified the sentence to read ‘The **annual** financial resources required to achieve these targets in low- and middle-income countries were estimated to increase **from about $16 billion in 2024** to just over $21.9 billion by 2030. (Figure 2).

21. Lines 223-224: “The largest amounts for direct services…” There seems to be a missing word (i.e., largest amount of what)?

**Response**: We have modified the sentence to read ‘The services requiring the most resources are ART (34% of total resource needs), prevention services for key populations (female sex workers (FSW), men who have sex with men (MSM), transgender people (TG), PWID) (13%), societal enabler interventions (10%).’

22. Lines 224-227: It is unclear what the denominator is for these %’s.

**Response**: See response to previous point.

23. Lines 232-236: It is unclear what “needs” is referring to. Is it the proportion of the $21.9 billion 2030 budget?

**Response**: We have indicated that it is a percentage of needs for all LMIC.

24. Could the authors clarify whether ARV and ART are used interchangeably or whether there is a distinction between the two terms in the manuscript? If the former, it would be helpful to pick one and remain consistent throughout the manuscript. If the latter, it would be helpful to specify what the distinction is in the manuscript.

**Response**: ARV and ART are used differently. ARV refers to just the drugs. ART refers to the complete service which include drugs, service delivery, labs, etc. We have added the definitions at first use of the abbreviation.

25. Line 267: It is unclear whether “total needs” is referring to the 2030 budget or something else.

**Response**: We modified the sentence to state ‘total financial needs in 2030’

26. Line 288: It is unclear whether Bulgaria and the Russian Federation were excluded from the study altogether or if they were simply excluded from either LMIC or high-income countries analyses?

**Response**: They were excluded from all the resource needs calculations since they are no longer LMICs. We mention this only because they were included in the 2020 round of estimates.

27. Lines 305-307: This seems to be an incomplete sentence?

**Response**: The last sentence was missing a period. That has been corrected.
