# Supplementary material for: The impact and cost of reaching the UNAIDS global HIV targets
Source: PLOS Glob Public Health. 2026 Oct 20;6(10):e0007360. doi: 10.1371/journal.pgph.0007360 (PMC13682660; doi:10.1371/journal.pgph.0007360)
Supplement: Peer review history

# Peer review: Decision Letter 0

**DOI:** 10.1371/journal.pgph.0007360.r001

**Authors:** Katherine Demi Kokkinias

1 Jun 2026

PGPH-D-25-03375

The impact and cost of reaching the UNAIDS global HIV targets

PLOS Global Public Health

Dear Dr. Stover,

Thank you for submitting your manuscript to PLOS Global Public Health. After careful consideration, we feel that it has merit but does not fully meet PLOS Global Public Health’s publication criteria as it currently stands. Therefore, we invite you to submit a revised version of the manuscript that addresses the points raised during the review process.

==============================

Please note that we have only been able to secure a single reviewer to assess your manuscript. We are issuing a decision on your manuscript at this point to prevent further delays in the evaluation of your manuscript. Please be aware that the editor who handles your revised manuscript might find it necessary to invite additional reviewers to assess this work once the revised manuscript is submitted. However, we will aim to proceed on the basis of this single review if possible.

The reviewer raised a number of major concerns.  They request additional information on several methodological aspects of the study (such as the thorough descriptions of the HIV simulation model, data sources used, and definitions of variables), interpretation of levels of data, and given the modeling assumptions, they recommend a sensitivity analysis be included. They also include several suggestions to improve grammar and overall clarity of the manuscript.

Could you please carefully revise the manuscript to address all comments raised?

==============================

Please submit your revised manuscript by Jul 27 2026 11:59PM. If you will need more time than this to complete your revisions, please reply to this message or contact the journal office at globalpubhealth@plos.org. When you're ready to submit your revision, log on to https://www.editorialmanager.com/pgph/ and select the 'Submissions Needing Revision' folder to locate your manuscript file.

Please include the following items when submitting your revised manuscript:

- A letter that responds to each point raised by the editor and reviewer(s). You should upload this letter as a separate file labeled 'Response to Reviewers'.
- A marked-up copy of your manuscript that highlights changes made to the original version. You should upload this as a separate file labeled 'Revised Manuscript with Track Changes'.
- An unmarked version of your revised paper without tracked changes. You should upload this as a separate file labeled 'Manuscript'.

Guidelines for resubmitting your figure files are available below the reviewer comments at the end of this letter.

As the corresponding author, your ORCID iD is verified in the submission system and will appear in the published article. PLOS supports the use of ORCID, and we encourage all coauthors to register for an ORCID iD and use it as well. Please encourage your coauthors to verify their ORCID iD within the submission system before final acceptance, as unverified ORCID iDs will not appear in the published article. *Only*  the individual author can complete the verification step; PLOS staff *cannot*  verify ORCID iDs on behalf of authors.

We look forward to receiving your revised manuscript.

Kind regards,

Katherine Demi Kokkinias, Ph.D.

Staff Editor

PLOS Global Public Health

Journal Requirements:

1. Please note that PLOS Global Public Health has specific guidelines on code sharing for submissions in which author-generated code underpins the findings in the manuscript. In these cases, we expect all author-generated code to be made available without restrictions upon publication of the work. Please review our guidelines at https://journals.plos.org/globalpublichealth/s/materials-and-software-sharing#loc-sharing-code and ensure that your code is shared in a way that follows best practice and facilitates reproducibility and reuse.
2. Please send a completed 'Competing Interests' statement, including any COIs declared by your co-authors. If you have no competing interests to declare, please state "The authors have declared that no competing interests exist". Otherwise please declare all competing interests beginning with the statement "I have read the journal's policy and the authors of this manuscript have the following competing interests:"
3. Please amend your detailed Financial Disclosure statement. This is published with the article. It must therefore be completed in full sentences and contain the exact wording you wish to be published.
  a. State the initials, alongside each funding source, of each author to receive each grant.
  b. State what role the funders took in the study. If the funders had no role in your study, please state: “The funders had no role in study design, data collection and analysis, decision to publish, or preparation of the manuscript.”
4. In the online submission form, you indicated that “Workbooks with all data and calculations are available from the authors.”.
  All PLOS journals now require all data underlying the findings described in their manuscript to be freely available to other researchers, either
  1. In a public repository,
  2. Within the manuscript itself, or
  3. Uploaded as supplementary information.
  This policy applies to all data except where public deposition would breach compliance with the protocol approved by your research ethics board. If your data cannot be made publicly available for ethical or legal reasons (e.g., public availability would compromise patient privacy), please explain your reasons by return email and your exemption request will be escalated to the editor for approval. Your exemption request will be handled independently and will not hold up the peer review process, but will need to be resolved should your manuscript be accepted for publication. One of the Editorial team will then be in touch if there are any issues.

If the reviewer comments include a recommendation to cite specific previously published works, please review and evaluate these publications to determine whether they are relevant and should be cited. There is no requirement to cite these works unless the editor has indicated otherwise.

Additional Editor Comments (if provided):

[Note: HTML markup is below. Please do not edit.]

Reviewers' comments:

Reviewer's Responses to Questions

**Comments to the Author**

1. Does this manuscript meet PLOS Global Public Health’s publication criteria?

Reviewer #1: Yes

**********

2. Has the statistical analysis been performed appropriately and rigorously?

Reviewer #1: N/A

**********

3. Have the authors made all data underlying the findings in their manuscript fully available (please refer to the Data Availability Statement at the start of the manuscript PDF file)?

The PLOS Data policy

Reviewer #1: Yes

**********

4. Is the manuscript presented in an intelligible fashion and written in standard English?

Reviewer #1: Yes

**********

Reviewer #1: This manuscript reports the health impact and costs of reaching the UNAIDS global HIV prevention and treatment targets by 2030, based on the model projections. Global HIV epidemic control is heavily dependent on international and domestic resources, but more international donors are reducing their financial assistance in recent years, making it harder for counties to continue combating HIV epidemic. The authors’ efforts to demonstrate health impact and cost of achieving those targets could help understand resource needs and potential means to better allocate limited resources.

Major comments:

1. Modeling methods and perspective: The analysis was based on the Goals model, potentially a micro-simulation model of cohorts with various population characteristics. It was not clearly described in the methods, and too brief in the abstract. Although a previous paper was cited, a succinct description about the model and analysis platform/software would be helpful for readers to follow your analysis. The presentation was aggregated in that 2030 UNAIDS targets were presented based on 134 country-level results. So, it certainly involved several layers of aggregation, i.e., county-region-global. Overall resource allocation relates to global funding whereas the local resource mobilization should be based on country level data. This brings some confusion about analysis perspective, which guides to what costs to include or exclude in the analysis. Unit costs used in the analysis may not be a true reflection of overall resources needs, if the costs excluded country-level administrative overhead and donor overheads; it was not clear how those costs were handled in the analysis.

2. Interventions and results: Not clear if it was an optimization model, and if the interventions can be prioritized, given the limited resources. Overall prioritization of the intervention from donors’ perspective may of interest, perhaps focus on key interventions showing high impacts. But country-level needs and domestic resource utilization and share of funding might lead to different prioritization. Although primary results were presented at a global level, you may focus on regional level results for details and stratifications (e.g., Figure 3); country-level results can be included in the appendix and would be helpful.

3. Uncertainty and sensitivity analysis: The modeling analysis and projections involved many input parameters and data/modeling assumptions; thus, uncertainty analysis should be an integral part of the presentation. The epidemiological and cost data were derived from various sources, and often they were assumed to be the same across counties and regions. Some basic sensitivity and scenario analyses may be used to address some of these issues.

Minor comments:

1. Line 26: including year of the Task Team, 2024 (?), would be useful.

2. Line 28: Methods presentation was very brief; some details would help readers, not reading the full paper.

3. Line 41-42: complex sentence, harder to read and understand what is being described.

4. Line 43-44: just one sentence on cost projection! Need more balanced presentation, perhaps clear focus on resource/cost impacts. May separate US and $, or say US dollars?

5. Line 50-51: why separate $ from the number? Need consistency in presentation.

6. Line: 74: Unusual to cite table in the introduction! It may be included in the methods.

7. Line 78: May start the sentence with 2024 Taks Team meeting, rather than describing previously published paper in the starting sentence! Methods section may be condensed, and description may be focused on main components of the model.

8. Line 104-133: long description about gender-based violence, discrimination, access to justice, sounds very general, and may be moved to appendix. Perhaps interventions should be prioritized on testing, treatment, and viral suppression, the core components, although other social/cultural factors may play roles in successful implementation?

9. Line 136: ‘above-site level’ cost? a new term, what does this include and how it was defined?

10. Line 138: ‘Ratios of support services to direct intervention spending’ sounds like a major part of the methods, but not clearly defined.

11. Line 148: unit cost description could be condensed, and perhaps include input table; online appendices are helpful too.

12. Line 82: may cite Table or Figure with results.

13. Line 130: ‘From 2010 to 2030’? Not clear why the projection had to go that far back!

14. Line 218: ‘Commodities’ sounds very generic, can use specific term, e.g., tests, medicines/drugs, and materials?

15. Line 222: ‘Target needs’ sounds different from resource needs described above, and here you have regional stratification; as noted in the major comments, this could be a major thread to present results. Also, providing sub-headings for major sections might help guide readers.

16. Line 268: a period missing after index.

17. Line 273-274: “foundation for countries to benchmark their current and projected

18. 274 domestic allocations”, this suggests country-level projections, and that may be included in the appendix. Also, this brings the question about mixing perspectives of donor vs countries domestic resource allocation, costs, and priorities.

19. Line 295: very few references listed (n=11), undermining the available literature and contributions of others in the field! It shows the literature review on interventions, methods, economic analysis, etc., not be comprehensive!

20. Figures and captions not clear, e.g., Figure 2 has many acronyms not defined, to make them independent of the text.

21. The following pre-print article may be of interest, showing program-level impact of funding reduction.

Reference:

The impact of the United States foreign aid freeze on HIV service delivery in PEPFAR-supported countries: a facility-level analysis of 2024–2025 programme data

https://www.medrxiv.org/content/10.64898/2026.04.17.26351143v1?ct=

**********

what does this mean?). If published, this will include your full peer review and any attached files.

**Do you want your identity to be public for this peer review?**  If you choose “no”, your identity will remain anonymous but your review may still be made public.

For information about this choice, including consent withdrawal, please see our Privacy Policy

Reviewer #1: No

**********

[NOTE: If reviewer comments were submitted as an attachment file, they will be attached to this email and accessible via the submission site. Please log into your account, locate the manuscript record, and check for the action link "View Attachments". If this link does not appear, there are no attachment files.]

# Author comment: Author response to Decision Letter 1

**DOI:** 10.1371/journal.pgph.0007360.r002

17 Jun 2026

Attachment Submitted filename: Response to reviewer comments June 17.docx

# Peer review: Decision Letter 1

**DOI:** 10.1371/journal.pgph.0007360.r003

**Authors:** Sharmistha Mishra

17 Aug 2026

PGPH-D-25-03375R1

The impact and cost of reaching the UNAIDS global HIV targets

PLOS Global Public Health

Dear Dr. Stover,

Thank you for submitting your manuscript to PLOS Global Public Health. After careful consideration, we feel that it has merit but does not fully meet PLOS Global Public Health’s publication criteria as it currently stands. Therefore, we invite you to submit a revised version of the manuscript that addresses the points raised during the review process.

Thank you for the revised manuscript, which had been sent out for review by the peer-reviewers from the original review, and a 2nd reviewer. Please see below for the reviewer comments which I agree will help strengthen the paper.

Please submit your revised manuscript by Sep 13 2026 11:59PM. If you will need more time than this to complete your revisions, please reply to this message or contact the journal office at globalpubhealth@plos.org. When you're ready to submit your revision, log on to https://www.editorialmanager.com/pgph/ and select the 'Submissions Needing Revision' folder to locate your manuscript file.

Please include the following items when submitting your revised manuscript:

- A letter that responds to each point raised by the editor and reviewer(s). You should upload this letter as a separate file labeled 'Response to Reviewers'.
- A marked-up copy of your manuscript that highlights changes made to the original version. You should upload this as a separate file labeled 'Revised Manuscript with Track Changes'.
- An unmarked version of your revised paper without tracked changes. You should upload this as a separate file labeled 'Manuscript'.

Guidelines for resubmitting your figure files are available below the reviewer comments at the end of this letter.

As the corresponding author, your ORCID iD is verified in the submission system and will appear in the published article. PLOS supports the use of ORCID, and we encourage all coauthors to register for an ORCID iD and use it as well. Please encourage your coauthors to verify their ORCID iD within the submission system before final acceptance, as unverified ORCID iDs will not appear in the published article. *Only*  the individual author can complete the verification step; PLOS staff *cannot*  verify ORCID iDs on behalf of authors.

We look forward to receiving your revised manuscript.

Kind regards,

Sharmistha Mishra, M.D., Ph.D

Academic Editor

PLOS Global Public Health

Journal Requirements:

1. Please note that PLOS Global Public Health has specific guidelines on code sharing for submissions in which author-generated code underpins the findings in the manuscript. In these cases, we expect all author-generated code to be made available without restrictions upon publication of the work. Please review our guidelines at https://journals.plos.org/globalpublichealth/s/materials-and-software-sharing#loc-sharing-code and ensure that your code is shared in a way that follows best practice and facilitates reproducibility and reuse.

If the reviewer comments include a recommendation to cite specific previously published works, please review and evaluate these publications to determine whether they are relevant and should be cited. There is no requirement to cite these works unless the editor has indicated otherwise.

Please review your reference list to ensure that it is complete and correct. If you have cited papers that have been retracted, please include the rationale for doing so in the manuscript text, or remove these references and replace them with relevant current references. Any changes to the reference list should be mentioned in the rebuttal letter that accompanies your revised manuscript. If you need to cite a retracted article, indicate the article’s retracted status in the References list and also include a citation and full reference for the retraction notice.

Please ensure you have clearly stated all data sources used in your study with references where appropriate and or links to the underlying data.

Additional Editor Comments (if provided):

[Note: HTML markup is below. Please do not edit.]

Reviewers' comments:

Reviewer's Responses to Questions

**Comments to the Author**

Reviewer #1: All comments have been addressed

Reviewer #2: (No Response)

**********

publication criteria?

Reviewer #1: Yes

Reviewer #2: Yes

**********

3. Has the statistical analysis been performed appropriately and rigorously?

Reviewer #1: N/A

Reviewer #2: Yes

**********

4. Have the authors made all data underlying the findings in their manuscript fully available (please refer to the Data Availability Statement at the start of the manuscript PDF file)?

The PLOS Data policy

Reviewer #1: Yes

Reviewer #2: Yes

**********

5. Is the manuscript presented in an intelligible fashion and written in standard English?

Reviewer #1: Yes

Reviewer #2: Yes

**********

**Review Comments to the Author**

Reviewer #1: Revised manuscript improved greatly in terms of clarity and presentation, and reviewers’ comments are addressed.

Minor comments:

Figure labels were not clearly presented; e.g., Line 192-193, cited Figure 1a, 1b, but in Line 201, it was cited as Figure 1. Figure 2, presentation looks like a single bar, perhaps alternative presentation would be clearer for readers.

Reviewer #2: Given the current HIV funding landscape, this is a timely study comparing the costs/resources needed and epidemiological impacts of two scenarios: 1) keeping all intervention coverage at 2023 constant levels versus 2) linear scale up of each intervention from 2025 to target value by 2030. Results from this study can enforce a call to action (i.e., that we still need mobilize additional resources including substantial increases in HIV funding in order to decrease HIV/AIDS incidence and related deaths).

The manuscript could benefit from several clarifications prior to publication as detailed below:

Major comments

- The Methods section in the Abstract and main manuscript text could benefit from explicitly stating whether the study included LMIC only or LMIC and high income countries. In particular, in line 31, it seems like only 118 LMIC countries were included in the study; however, lines 47-48 quantify budget needed for high income countries as well. Further, lines 45-46 discuss the “resources needed to achieve the targets in all low- and middle-income countries (LMIC) would rise to US$21.9 billion by 2030); however, lines 47-48 state “of this [US$21.9 billion] amount…45% in upper-middle income countries.” It is unclear why the upper-middle income country budget would be reported as a proportion of the LMMIC budget. In line 101, it would be helpful to specify whether these 134 countries are LMIC or does it also include high income countries?

- The Methods would benefit from additional details about the Goals and Resource Needs Models. The manuscript currently states that these are “simulation models”. However, it is unclear whether these are microsimulation models, agent-based models, etc. Further, is it unclear whether these models are dynamic or static and whether indirect effects from the interventions were included in the models.

- Several sentences lack references (e.g., lines 66-67, lines 78-80, line 86, lines 120-121, lines 142-143, line 281). These line numbers are not an exhaustive list. The manuscript would benefit from the authors thoroughly checking and ensuring references are included where appropriate throughout the manuscript.

- Given donor funding cuts from PEPFAR and internationally, other modelling studies have modelled the impacts of reduced intervention coverage. (E.g., see: Ratevosian, J., Ngangula, P., & Tram, K. H. (2025). Modeling the fallout: projecting the global impact of donor funding cuts on HIV prevention, treatment, and care. Current Opinion in HIV & AIDS, 20(6), 621–631. https://doi.org/10.1097/COH.0000000000000977; Godbole, R., Wachinger, J., Nichols, C. et al. Shifts in PEPFAR-supported HIV treatment in 26 countries from 2024 to 2025. Nat. Health (2026). https://doi.org/10.1038/s44360-026-00159-1). The current manuscript could also benefit from modelling a decreased intervention coverage scenario in addition to the two scenarios modelled.

- The Methods would benefit from more details in costs including: 1) specifying what year costs were reported in (e.g., 2026 USD); and 2) specifying whether discounting and/or inflation were considered.

- Lines 263-266 state that, “An increase of 20% in the cost of ARVs in 2030 across all countries would raise the total resource needs by 4%. A similar increase in the unit costs of service delivery for treatment or laboratory tests would increase total needs by 4% and 2% respectively.” Originally, I understood the 20% increase in the cost of ARVs in 2030 to be due to the increased number of people on ARVs. However, the wording in the second sentence seems to imply that it is the unit cost of ARVs that are increasing resulting in a 20% increase in the cost of ARVs. If this is true, it would be helpful to 1) specify the assumed proportion increase in ARV unit costs in the Methods and Data section; and 2) explain why unit costs of ARVs are expected to increase (e.g., is the use of newer patented ARVs expected to outweigh the increased use and availability of generic ARVs?).

- Some parts of the Discussion do not flow from the Results. For example, lines 276-277 state that “estimates [from the current study] update previous work that estimated that new infections would fall to 370,000 new infections and 250,000 AIDS deaths by 2025.” It would be helpful to mention what results from the current study these findings are being compared to and discuss what the key factors responsible for the differences in results between the current study and the previous study are? Another example is lines 281-282 which state that the “price reductions observed for ARVs during the 2021-2024 period.” This seems to contradict previous results stating an increase in ARV costs. Could authors clarify?

- While a couple of limitations are mentioned in the Discussion, the Discussion could benefit from a Limitations section that discusses model limitations, data limitations, limitations due to assumptions, etc., and the impact of these limitations on study findings in more detail.

Minor comments

- Lines 25-26: Check grammar, capitalization, spacing.

- Line 46: LMIC could have been defined earlier in the abstract (i.e., line 31).

- Lines 66-68: It is unclear whether these statistics are global, for low and middle income countries, or for another region.

- Adding subheadings throughout the Methods would help improve readability.

- Lines 143-145: Check grammar

- Please ensure all acronyms (e.g., ARV) are defined upon first use.

- Line 158: Define what year “current” is referring to in “current procurement prices.”

- Lines 168-171: In addition to the absolute $ declined from current to 2030 prices, would be helpful to add a proportion (e.g., X% decline compared to current prices).

- Throughout the Results, it would be helpful to indicate that the numbers in brackets are 95% CI (e.g., lines 191-192).

- Line 194: Can authors confirm whether the decline from “40 (37-46) million to 39 (36-45) million” was a decline from 2023 to 2030?

- Lines 222-223 state that the “financial resources… were estimated to increase to just over $21.9 billion by 2030.” It is unclear what the comparator is (i.e., increase from when) and whether this number is referring to $21.9 billion/year for all included countries or $21.9 billion cumulative for all included countries.

- Lines 223-224: “The largest amounts for direct services…” There seems to be a missing word (i.e., largest amount of what)?

- Lines 224-227: It is unclear what the denominator is for these %’s.

- Lines 232-236: It is unclear what “needs” is referring to. Is it the proportion of the $21.9 billion 2030 budget?

- Could the authors clarify whether ARV and ART are used interchangeably or whether there is a distinction between the two terms in the manuscript? If the former, it would be helpful to pick one and remain consistent throughout the manuscript. If the latter, it would be helpful to specify what the distinction is in the manuscript.

- Line 267: It is unclear whether “total needs” is referring to the 2030 budget or something else.

- Line 288: It is unclear whether Bulgaria and the Russian Federation were excluded from the study altogether or if they were simply excluded from either LMIC or high-income countries analyses?

- Lines 305-307: This seems to be an incomplete sentence?

**********

what does this mean?). If published, this will include your full peer review and any attached files.

**Do you want your identity to be public for this peer review?**  If you choose “no”, your identity will remain anonymous but your review may still be made public.

For information about this choice, including consent withdrawal, please see our Privacy Policy

Reviewer #1: No

Reviewer #2: **Yes:**  Min Xi

**********

[NOTE: If reviewer comments were submitted as an attachment file, they will be attached to this email and accessible via the submission site. Please log into your account, locate the manuscript record, and check for the action link "View Attachments". If this link does not appear, there are no attachment files.]

# Author comment: Author response to Decision Letter 2

**DOI:** 10.1371/journal.pgph.0007360.r004

20 Aug 2026

Attachment Submitted filename: Response to reviewers.docx

# Peer review: Decision Letter 2

**DOI:** 10.1371/journal.pgph.0007360.r005

**Authors:** Lucy Chimoyi

7 Sep 2026

The impact and cost of reaching the UNAIDS global HIV targets

PGPH-D-25-03375R2

Dear Mr Stover

We are pleased to inform you that your manuscript 'The impact and cost of reaching the UNAIDS global HIV targets' has been provisionally accepted for publication in PLOS Global Public Health.

Before your manuscript can be formally accepted you will need to complete some formatting changes, which you will receive in a follow up email. A member of our team will be in touch with a set of requests.

Please note that your manuscript will not be scheduled for publication until you have made the required changes, so a swift response is appreciated.

IMPORTANT: The editorial review process is now complete. PLOS will only permit corrections to spelling, formatting or significant scientific errors from this point onwards. Requests for major changes, or any which affect the scientific understanding of your work, will cause delays to the publication date of your manuscript.

If your institution or institutions have a press office, please notify them about your upcoming paper to help maximize its impact. If they'll be preparing press materials, please inform our press team as soon as possible -- no later than 48 hours after receiving the formal acceptance. Your manuscript will remain under strict press embargo until 2 pm Eastern Time on the date of publication. For more information, please contact globalpubhealth@plos.org.

Thank you again for supporting Open Access publishing; we are looking forward to publishing your work in PLOS Global Public Health.

Best regards,

Lucy Chimoyi

Academic Editor

PLOS Global Public Health

***********************************************************

Reviewer Comments (if any, and for reference):

Reviewer's Responses to Questions

**Comments to the Author**

Reviewer #1: All comments have been addressed

Reviewer #2: All comments have been addressed

**********

publication criteria?

Reviewer #1: Yes

Reviewer #2: Yes

**********

3. Has the statistical analysis been performed appropriately and rigorously?

Reviewer #1: N/A

Reviewer #2: Yes

**********

4. Have the authors made all data underlying the findings in their manuscript fully available (please refer to the Data Availability Statement at the start of the manuscript PDF file)?

The PLOS Data policy

Reviewer #1: (No Response)

Reviewer #2: Yes

**********

5. Is the manuscript presented in an intelligible fashion and written in standard English?

Reviewer #1: Yes

Reviewer #2: Yes

**********

Reviewer #1: They were minor comments; all addressed.

Reviewer #2: Thank you for the opportunity to re-review this work. All previous comments have been addressed; the revised manuscript is strengthened in terms of clarity and cohesiveness.

Minor comments:

- ANC seems to be defined multiple times throughout the manuscript. It only needs to be defined once.

- CI should be defined as confidence interval in line 206.

**********

what does this mean?). If published, this will include your full peer review and any attached files.

**Do you want your identity to be public for this peer review?**  If you choose “no”, your identity will remain anonymous but your review may still be made public.

For information about this choice, including consent withdrawal, please see our Privacy Policy

Reviewer #1: No

Reviewer #2: **Yes:**  Min Xi

**********
